# Possible relationship between common genetic variation and white matter development in a pilot study of preterm infants

**DOI:** 10.1002/brb3.434

**Published:** 2016-04-02

**Authors:** Michelle L. Krishnan, Zi Wang, Matt Silver, James P. Boardman, Gareth Ball, Serena J. Counsell, Andrew J. Walley, Giovanni Montana, Anthony David Edwards

**Affiliations:** ^1^ Centre for the Developing BrainKing's College LondonSt Thomas' HospitalLondonSE1 7EHUK; ^2^ Department of Biomedical EngineeringKing‘s College LondonSt Thomas‘ HospitalLondon SE1 7EHUK; ^3^ Department of Population HealthLondon School of Hygiene and Tropical MedicineLondonWC1E 7HTUK; ^4^ MRC Centre for Reproductive HealthUniversity of EdinburghEdinburghEH16 4TJUK; ^5^ School of Public HealthFaculty of MedicineImperial College LondonNorfolk PlaceLondonW2 1PGUK

**Keywords:** Brain development, genomics, lipids, magnetic resonance imaging, metabolic pathways, multivariate analysis, neonatal, single‐nucleotide polymorphism

## Abstract

**Background:**

The consequences of preterm birth are a major public health concern with high rates of ensuing multisystem morbidity, and uncertain biological mechanisms. Common genetic variation may mediate vulnerability to the insult of prematurity and provide opportunities to predict and modify risk.

**Objective:**

To gain novel biological and therapeutic insights from the integrated analysis of magnetic resonance imaging and genetic data, informed by prior knowledge.

**Methods:**

We apply our previously validated pathway‐based statistical method and a novel network‐based method to discover sources of common genetic variation associated with imaging features indicative of structural brain damage.

**Results:**

Lipid pathways were highly ranked by Pathways Sparse Reduced Rank Regression in a model examining the effect of prematurity, and PPAR (peroxisome proliferator‐activated receptor) signaling was the highest ranked pathway once degree of prematurity was accounted for. Within the PPAR pathway, five genes were found by Graph Guided Group Lasso to be highly associated with the phenotype: aquaporin 7 (AQP7), malic enzyme 1, NADP(+)‐dependent, cytosolic (ME1), perilipin 1 (PLIN1), solute carrier family 27 (fatty acid transporter), member 1 (SLC27A1), and acetyl‐CoA acyltransferase 1 (ACAA1). Expression of four of these (ACAA1, AQP7, ME1, and SLC27A1) is controlled by a common transcription factor, early growth response 4 (EGR‐4).

**Conclusions:**

This suggests an important role for lipid pathways in influencing development of white matter in preterm infants, and in particular a significant role for interindividual genetic variation in PPAR signaling.

## Introduction

Preterm birth accounts for 11% of all births (Blencowe et al. 2012), and is the leading global cause of deaths under 5 years of age (March of Dimes, 2012; World Health Organization, 2014). Over 30% of survivors experience cognitive problems (Moore et al. 2012) which last into adulthood, manifesting in a specific manner with anxiety, inattention, and social and communication problems compared to term‐born infants (Hack 2009). This is associated with a higher prevalence of psychopathology with a three to eightfold increased risk of behavioral problems in preterm infants, and a three to eightfold increase in the prevalence of Autism Spectrum Disorders (ASD) compared to the general population (Baird et al. 2006; Williams et al. 2006; Johnson and Wolke 2013), as well as a risk ratio of 7.4 for bipolar affective disorder among infants born below 32 weeks of gestation (Nosarti et al. 2012).

White matter myelination in normal growth is typically restricted to the brain stem, globus pallidus and ventro‐lateral nucleus of the thalamus up to term (Brody et al. 1987; Kinney et al. 1988; Tanaka et al. 1995), then enters a period of rapid development from 38 weeks' gestation onwards. This continues apace particularly in the first and second years, and extends into adulthood (Yakovlev and LeCours 1967; Bartzokis et al. 2010; Groeschel et al. 2010; Miller et al. 2012). White and gray matter show linked yet characteristic trajectories in normal development (Groeschel et al. 2010), with brain volume, cortical thickness, and surface area peaking in growth rate during late childhood/early adolescence (Lenroot et al. 2007; Nie et al. 2013), and cortical folding seeming to peak earlier in childhood (Zilles et al. 1989; Armstrong et al. 1995; Nie et al. 2013; Li et al. 2014). The effect of prematurity on these processes as evaluated with MRI indicates widespread alterations of the white matter that correlate with functional measures (Krishnan et al. 2007; Counsell et al. 2008; Eikenes et al. 2011; van Kooij et al. 2012; Groppo et al. 2014), paralleled by changes in the overlying gray matter (Ajayi‐Obe et al. 2000; Ball et al. 2013; Vinall et al. 2013; Smyser et al. 2015).

Preterm brain injury can be considered a broad entity resulting from various factors such as hypoxia, ischemia, infection, and inflammation (Volpe 2009; Jablonska et al. 2012) that can have a variable impact on development. The principal neuropathological mechanisms in the preterm infant have been identified as periventricular leukomalacia (PVL) and neuronal/axonal disease, forming the composite of encephalopathy of prematurity. PVL is most commonly of a diffuse form and involves microscopic necroses that later form glial scars which are hard to detect with imaging (Volpe 2009). The more visible aspect of PVL comprises marked astrogliosis and microgliosis, alongside fluctuations in numbers of premyelinating oligodendrocytes and oligodendroglial progenitors (Haynes et al. 2003; Back et al. 2005; Robinson et al. 2006; Billiards et al. 2008).

Diffusion MRI (d‐MRI) provides measures of white matter structure that are correlated with neurodevelopmental outcome (Counsell et al. 2008; van Kooij et al. 2012; Ball et al. 2015) and highly heritable, such that 60% of the variability in d‐MRI measures between individuals in the neonatal period can be attributed to genetic factors and persists into adulthood (Geng et al. 2012; Shen et al. 2014). The d‐MRI measure of fractional anisotropy FA (the fraction of the magnitude of diffusion that can be attributed to directionally dependent diffusion), has been shown to increase during the early (premyelination) stage of white matter development (Wimberger et al. 1995) and subsequent myelination by maturing oligodendrocytes leads to further increases in FA, with early sites including the posterior limb of the internal capsule and the optic radiations (Huppi et al. 1998; Neil et al. 1998; Drobyshevsky et al. 2005). Decreased fractional anisotropy FA in preterm infants is related to cognitive, fine‐motor, and gross‐motor outcome at 2 years (van Kooij et al. 2012), and these alterations of white matter persist into adulthood in very preterm individuals and are associated with cognitive function (Allin et al. 2011). Imaging endophenotypes provide a more direct link to genetic underpinnings than the neurodevelopmental or behavioral features of disease, demonstrating higher genetic penetrance and informing on the biological foundation of disease.

Susceptibility to perinatal brain injury is likely to be modulated by the combined effects of multiple genes of individually small effect in response to environmental influences during pregnancy and in the early postnatal period (Dempfle et al. 2008; Leviton et al. 2015). Common DNA sequence variation is estimated to account for up to 50% of additive genetic variation in complex traits, including neuroanatomical features (Yang et al. 2010; Toro et al. 2014) as well as neurological disorders including autism (Gaugler et al. 2014), epilepsy (Speed et al. 2014), and schizophrenia (Arnedo et al. 2014). Given that preterm birth poses an extreme challenge to the whole organism, it is plausible that common variation between individuals results in differential vulnerability to adverse stimuli, impacting development.

In this work, we focus on understanding the influence of common genetic variation on white matter development within the preterm population. The stress of premature extrauterine life leads to a broad range of neuroimaging changes and related neurological outcomes, producing contrast between individuals that we are able to use here in a within‐group design. The sparse regression approaches used in this study have been developed for the selection of explanatory variables associated with a quantitative trait within a study cohort, and we employ these methods here for that purpose.

Statistical genetics and bioinformatics methods allow the principled joint analysis of the large imaging and genetic datasets involved, and facilitate the biological interpretation of results. In contrast with a hypothesis‐based approach that aims to test a specific set of assumptions against a significance threshold, a data‐driven technique seeks a principled and biologically informed way to uncover the signal within the data, yielding unbiased and novel insights that can be validated experimentally in a constructive and iterative manner (Robinson et al. 2014). A traditional hypothesis‐based method is the genome‐wide association study (GWAS) whereby each single‐nucleotide polymorphism (SNP) is tested for association with the phenotype, requiring typically hundreds of thousands of hypotheses and stringently adjusted p‐values. The regression modeling approach, in comparison, involves fitting a predictive model for the phenotype using all SNPs, while also ranking all SNPs based on their predictive value. Regression modeling is of particular benefit in imaging genomics studies where the space of possible hypotheses is vast, encompassing genetic features (SNPs) multiplied by image features (voxels). This obviates the need for multiple‐testing correction and significance thresholds, while producing a meaningful ranking of results (Silver and Montana 2012b).

In these large datasets, the number of subjects *n* is typically much smaller than the number of features *p* (e.g., single‐nucleotide polymorphisms SNPs), posing a statistical and analytical problem. Two current approaches are to either increase *n* significantly or find a principled way to reduce *p* while preserving the underlying signal. We have addressed this problem by developing a pathways‐driven sparse regression method (PsRRR) (Silver and Montana 2012b) which we have robustly validated (Silver et al. 2013) and extended to multivariate imaging traits (Silver et al. 2012a). We have subsequently applied the Graph Guided Group Lasso (GGGL) to improve SNP and gene selection by integrating information from grouping SNPs into genes and organizing genes into a weighted gene network encoding the functional relatedness between all pairs of genes (Wang and Montana 2014). We apply these methods to the preterm population, leveraging prior biological knowledge by using SNPs and genes grouped into biological pathways or networks, which allows the detection of previously unexposed signal (Wang et al. 2010) and eases the interpretation of results (Cantor et al. 2010). Common genetic variation within biological canonical pathways and functional networks is used to explain interindividual variation in imaging features relevant to neurodevelopmental outcome.

## Patients and Methods

### Patient characteristics

Participants' characteristics: mean GA 28 + 4 weeks, range 23 + 2 to 32 + 6, mean PMA at scan 40 + 3, range 27 + 4 to 47 + 6 weeks. This cohort has previously been described in detail (Boardman et al. 2014). Research was carried out in compliance with the Code of Ethics of the World Medical Association (Declaration of Helsinki), with approval from the NHS Research Ethics Committee and to the standard of the associated granting agencies.

### MR Image acquisition and analysis

MR images were acquired for 72 preterm infants (mean gestational age (GA) 28 + 4 weeks, mean postmenstrual age (PMA) at scan 40 + 3 weeks). Imaging was performed on a Philips 3‐Tesla system (Philips Medical Systems, Netherlands) using an eight‐channel phased array head coil. Single‐shot echo‐planar diffusion tensor imaging was acquired in the transverse plane in 15 noncollinear directions using the following parameters: repetition time (TR): 8000 msec; echo time (TE): 49 msec; slice thickness: 2 mm; field of view: 224 mm; matrix: 128 × 128 (voxel size: 1.7531 × 1.753 × 2 mm^3^); *b* value: 750 sec/mm^2^; SENSE factor: 2. T1‐weighted 3D MPRAGE were acquired with parameters: TR = 17 msec, TE = 4.6 msec, inversion delay = 1500 msec, flip angle = 13°, acquisition plane = sagittal, voxel size = 0.82 × 1.03 × 1.6 mm, FOV = 210 ×167 mm and acquired matrix = 256 × 163. T2‐weighted fast spin‐echo: TR × 8700 ms, TE = 160 msec, flip angle = 90°, acquisition plane = axial, voxel size = 1.15 × 1.18 × 2 mm, FOV = 220 mm, and acquired matrix = 192 × 186.

Diffusion tensor imaging (DTI) analysis was performed by using FMRIB's Diffusion Toolbox (v2.0; RRID:nif‐0000‐00305) as implemented in FMRIB's Software Library (FSL v4.1.5; www.fmrib.ox.ac.uk/fsl) (Smith and Nichols 2009). Each infant's diffusion‐weighted image (DWI) was registered to their respective nondiffusion‐weighted (*b* = 0) image and corrected for differences in spatial distortion owing to eddy currents. Images were brain extracted using Brain Extraction Tool (BET v2.1), diffusion tensors calculated voxelwise, and FA maps generated.

Tract‐Based Spatial Statistics (TBSS) (Smith et al., 2006) was performed by using a modified pipeline specifically optimized for neonatal DTI analysis. This included an initial low degrees‐of‐freedom linear registration step and a second registration to a population‐average FA map, which has been shown to reduce global misalignment between neonatal fractional anisotropy (FA) maps (Ball et al. 2010). The aligned data were used to create a mean FA map and a mean FA skeleton that represents the center of all white matter tracts common to the group. The FA skeleton was thresholded at FA ≥0.2 before each infant's aligned FA data were projected onto it.

Three separate adjustments for clinical variables were made to the TBSS phenotype using the FSL general linear model tool, with permutation‐based significance testing using the randomize tool with Threshold‐Free Cluster Enhancement. First, imaging data were adjusted for the effect of PMA at scan only; second, the imaging data were adjusted for both PMA and GA, thereby focusing on the effect of genetic variation and environment. Third, there was an adjustment for genetic ancestry. In all cases, dimension reduction in the phenotype with principal component analysis was carried out for computational efficiency.

### Saliva genotyping

The concentration of all the genomic DNA samples was measured using the PicoGreen protocol. 200 ng of genomic DNA was used for each Illumina HumanOmniExpress‐12 array according to the manufacturer's instructions. HumanOmniExpress‐12 arrays have 730,525 markers with a mean spacing of ~4 kb. 392,197 of those markers are within 10 kb of a known RefSeq gene and there are 15,062 coding SNPs and 7459 MHC markers included in that total. All samples successfully passed quality control.

### Genome‐wide genotyping

Samples were genotyped on Illumina HumanOmniExpress‐12 arrays. The genotype matrix was recoded in terms of minor allele counts, including only SNPs with MAF ≥5% and ≥99% genotyping rate (Purcell et al. 2007). After these filtering steps, 613,186 SNPs remained.

### Assessment of population stratification

Whole genome SNP data were used for complete linkage agglomerative clustering, based on pairwise identity‐by‐state (IBS) distance as implemented in PLINK 1.9 (Chang et al. 2015), to assess whether any two individuals belonged to the same population. Dimension reduction in the IBS distance matrix was carried out by principal component analysis, and the first principal component was used as a covariate in TBSS analysis to adjust for population stratification.

Information on self‐reported ethnicity (as defined in ISB standard DSCN 11/2008) was collected by asking mothers (and fathers when present) to define themselves according to a list of options. The terms were drawn from Ethnic Category National Codes as in Department of Health Guidance at the time. Parental self‐reported ethnicity was summarized into broader categories for the purposes of data visualization by aggregating all “White” subcategories into a single group “White”, all “Black” subcategories into “Black”, and all “Asian” subcategories into “Asian”. In cases where either one parent self‐reported as “Mixed” or if there was a discrepancy between maternal and paternal ethnicities, the term “Mixed” was applied. Where parents were both from an ASEAN member state (two cases) the individual was classified by the authors as “SE Asian”. These aggregated ethnic categories were used to label the datapoints of the PCA plot of the first two principal components of the IBS variance‐standardized relationship matrix. This illustrates the correspondence between the first two components of genetic ancestry and ethnicity, and provides an overview of the cohort population mixture.

### Pathways sparse reduced rank regression

Pathways sparse reduced rank regression estimates the regression coefficients in the linear model with multivariate responses, subjected to constraints. The model accounts for potential biasing factors such as pathway linkage disequilibrium and size by using an adaptive, weight‐tuning procedure. Pathway weightings in the regression model are adjusted according to the empirical bias in pathway selection frequencies, obtained by fitting the PsRRR model with a null response. Depending on the degree of penalization, some coefficients are driven to zero, thus performing variable selection so that only SNPs within associated pathways are retained in the model. Pathways are ranked in order of importance using a resampling strategy, with highest ranked pathways having highest selection frequency and highest correlation with the phenotype. SNPs were mapped to genes (NCBI GRCh37) and KEGG pathways (Kyoto Encyclopedia of Genes and Elements), after excluding cancer‐related pathways due to high redundancy in gene membership as previously described (Silver et al. 2012a), which nonetheless allows the genes to participate in the model as part of other pathways. Parameters used: Model adjusted for PMA; *λ* 0.99, 100 subsamples, 20 iterations with 2000 × 10 plus 4000 × 10 model fits per iteration. Model adjusted for GA and PMA; *λ* 0.99, 100 subsamples, 20 iterations with 2000 × 10 plus 4000 × 10 model fits per iteration.

### Graph Guided Group Lasso

Graph Guided Group Lasso incorporates prior information from SNP‐gene mapping as well as from the gene functional interaction network to guide variable selection (Wang and Montana 2014). The functional relationships between genes within the top ranked pathway peroxisome proliferator‐activated receptor (PPAR) signaling pathway were systematically described by clustering the genes based on their GO BP annotations (Ovaska et al. 2008). This resulted in an adjacency matrix based on pairwise semantic similarity of GO terms, which has been shown to correlate with protein sequence similarity (Apweiler et al. 2004) and protein family similarity (Couto et al. 2006).

The GGGL approach assumes a linear relationship between gene function (described here by GO annotation) and brain imaging features, applying an additional penalty function on the regression coefficients that incorporates network structure information. The model selects functionally related genes that are associated with the trait and identifies important SNPs within the selected genes. GGGL‐1 further imposes that SNPs in functionally related genes have similar effects on the phenotypic trait.

The regularization parameter *μ* controls the weight of the prior knowledge added to the squared loss function. When *μ* is large the model relies more on prior knowledge from the network. Using GGGL‐1 with *μ* = 0.1, 1, and 10 resulted in the same set of genes with selection probability > 0.4 (a threshold representing a step change in the probability distribution). Using GGGL‐2 with *μ* = 0.1, 1, 10 also yielded very stable results, with 27 of the top 30 SNPs appearing in all three lists for different values of *μ*, and the same genes were selected in the three instances. Both versions of the method (GGGL‐1 and GGGL‐2) produced comparable results, with the same genes and SNPs being ranked highly by both approaches (Tables S8 and S9). Genes with more than one occurrence and a selection probability threshold *>*0.4 were retained, at a threshold representing a step change in the probability distribution.

## Results

### Imaging phenotypes

A group white matter skeleton was constructed and used to extract voxel‐wise fractional anisotropy (FA) values for each of the 72 individuals. These values were serially adjusted for important clinical variables: effect of gestational age (GA) at birth, effect of postmenstrual age (PMA) at scan and genetic ancestry, resulting in three separate phenotypes. In all instances the phenotypes underwent dimension reduction with principal component analysis (PCA). The elbow of the scree plot was selected in each case to determine the number of components used for the subsequent analysis (scree plots in Figure S1).

**Figure 1:**
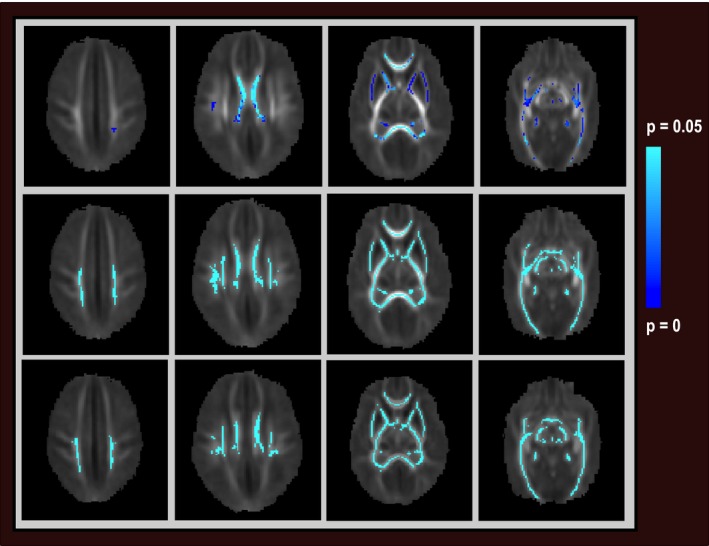
Group white matter DTI skeleton, showing voxels that vary significantly between individuals (corrected *P* < 0.05 for all voxels, darker blue signifies lower *P*‐value). Axial views superior to inferior left to right. Top row: Voxels varying between individuals adjusting for PMA at scan. Middle row: Voxels varying between individuals adjusting for GA at birth and PMA. Bottom row: Voxels varying between individuals adjusting for GA, PMA and genetic ancestry.

The statistic images (Fig. 1) indicate that GA at birth had a significant impact on the central white matter in particular, whereas including correction for PMA resulted in a more diffuse effect, and correcting for genetic ancestry in addition to GA and PMA had little effect on the phenotype (distributions of residuals in Figure S2).

### Effect of population stratification

We use the term population stratification to refer here to allele frequency differences between subpopulations within a population. Genetic ancestry is a cause of population stratification, and the term here refers to the use of empirical methods to assign an ancestry classification (Ali‐Khan et al. 2011). Population stratification of the cohort was assessed by calculating pairwise identity by state (IBS) values and using these to perform complete linkage clustering (Methods). This revealed a degree of stratification along the first two components, corresponding with parental self‐reported ethnicity (Figure S3).

When the effect of genetic ancestry was included in the imaging phenotype, the PsRRR algorithm did not converge, suggesting a loss of signal in the relationship between genetic predictors and imaging phenotype after adjustment for genetic ancestry. Subsequent analyses therefore refer only to the models adjusting for GA and/or PMA.

### Pathway ranking

SNPs were mapped to genes and pathways from the Kyoto Encyclopedia of Genes and Elements (KEGG) as summarized in Table S4.

**Figure 2:**
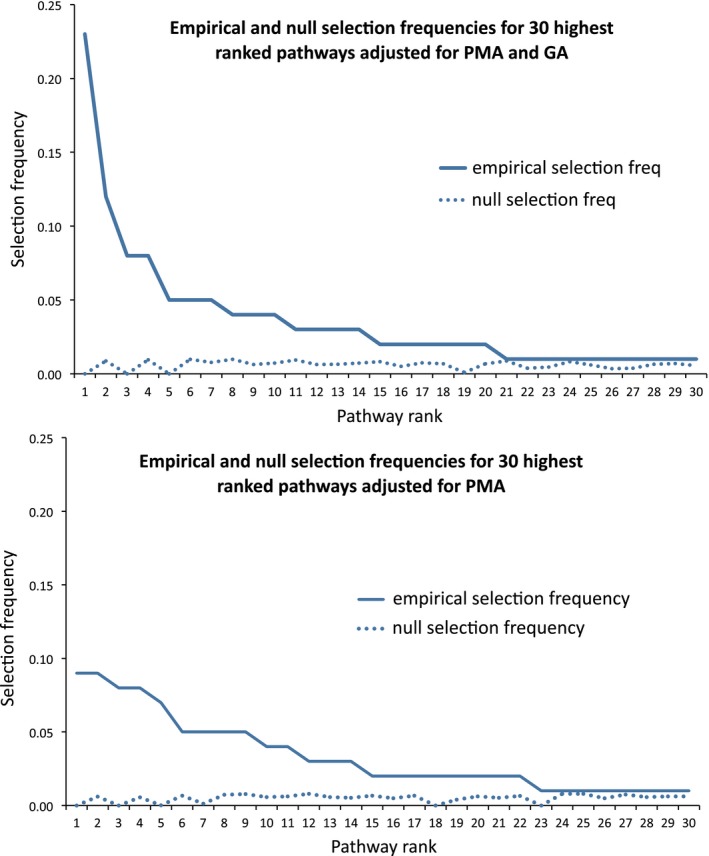
Empirical and null selection frequencies for thirty most predictive pathways, adjusted for PMA (top) and adjusted for GA and PMA (bottom).

The PsRRR method was used to identify and rank biological pathways predictive of white matter integrity. Pathways were ranked by stability selection in order of selection frequency, with each adjusted phenotype analyzed in turn (Table 1). These empirical results were compared with null selection frequencies (Fig. 2, Figure S5, Table S6, and Table S7).

**Table 1:** Top thirty KEGG pathways ranked by PsRRR. Lipid pathways highlighted in bold. Left column: Results with phenotype adjusted for PMA. Right column: Results adjusted for GA and PMA

Pathways adjusted for PMA	Pathways adjusted for GA and PMA
**Glycine Serine and Threonine Metabolism**	**PPAR Signaling Pathway**
**PPAR Signaling Pathway**	Dilated Cardiomyopathy
**Alpha‐Linolenic Acid Metabolism**	**Glycerolipid Metabolism**
**Ether Lipid Metabolism**	**Alpha‐Linolenic Acid Metabolism**
**Glycerophospholipid Metabolism**	Pyrimidine Metabolism
SNARE Interactions In Vesicular Transport	Calcium Signaling Pathway
Hypertrophic Cardiomyopathy HCM	Cardiac Muscle Contraction
**Glycerolipid Metabolism**	Hematopoietic Cell Lineage
Basal Transcription Factors	Complement and Coagulation Cascades
Cardiac Muscle Contraction	Aminoacyl tRNA Biosynthesis
Hematopoietic Cell Lineage	Pancreatic Cancer
**Phosphatidylinositol Signaling System**	Renin Angiotensin System
Ubiquitin Mediated Proteolysis	Nucleotide Excision Repair
Nucleotide Excision Repair	SNARE Interactions in Vesicular Transport
JAK‐STAT Signaling Pathway	**Glycosylphosphatidylinositol GPI Anchor Biosynthesis**
**Adipocytokine Signaling Pathway**	Type II Diabetes Mellitus
**Glycosylphosphatidylinositol GPI Anchor Biosynthesis**	Epithelial Cell Signaling in Helicobacter Pylori Infection
GNRH Signaling Pathway	DNA Replication
Starch and Sucrose Metabolism	Glycine Serine and Threonine Metabolism
Long‐Term Depression	Sulfur Metabolism
ABC Transporters	Dorso Ventral Axis Formation
Endocytosis	Peroxisome
**Fatty Acid Metabolism**	Bladder Cancer
Antigen Processing and Presentation	Primary Immunodeficiency
Ascorbate and Aldarate Metabolism	Ascorbate and Aldarate Metabolism
Lysosome	Lysosome
One Carbon pool by Folate	One Carbon pool by Folate
Fc Epsilon RI Signaling Pathway	Axon Guidance
Viral Myocarditis	Vascular Smooth Muscle Contraction
Complement and Coagulation Cascades	Fc Epsilon RI Signaling Pathway

PMA, postmenstrual age; GA, gestational age; PAPR, peroxisome proliferator‐activated receptor; KEGG, Kyoto Encyclopedia of Genes and Elements; PsRRR, pathways‐driven sparse regression method

**Figure 3:**
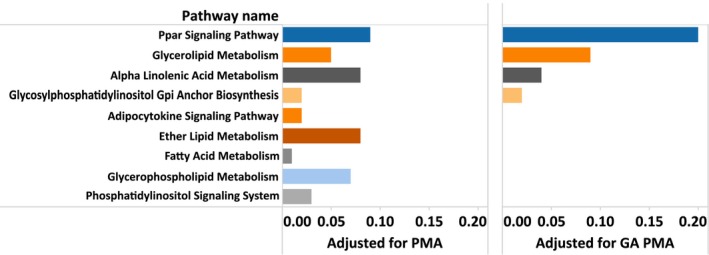
Empirical selection frequencies for lipid pathways among top thirty ranked by PsRRR, sorted by rank postadjustment for GA and PMA. Left: adjusted by postmenstrual age (PMA); Right: Adjusted for gestational age (GA) and postmenstrual age (PMA).

Lipid pathways were significantly over‐represented in the top ranking pathways adjusted for PMA (*P* ≤ 0.005) (Table 1, bold) and although the total number of lipid pathways decreased with adjustment for GA, the empirical selection frequency of the most highly ranked lipid pathway (peroxisome proliferator‐activated receptor (PPAR) signaling) increased from 0.09 to 0.2 (Fig. 3).

### Gene relationships

The GGGL method was applied to genes in the most highly ranked KEGG pathway (PPAR signaling) (Patients and Methods). This allowed clarification of the association between individual genes and SNPs with the phenotype, which was constrained to a biological pathway of interest. Within the PPAR pathway, the GGGL‐1 method selected a subset of genes (5/69) functionally related in terms of Gene Ontology (GO) Biological Process (BP) and linearly correlated with white matter FA. These were aquaporin 7 (AQP7), malic enzyme 1, NADP(+)‐dependent, cytosolic (ME1), perilipin 1 (PLIN1), solute carrier family 27 (fatty acid transporter), member 1 (SLC27A1), and acetyl‐CoA acyltransferase 1 (ACAA1). Of the top thirty SNPs selected by the GGGL‐2 method, all were found within genes with selection probability >0.4 in GGGL‐1, indicating strong agreement between the two approaches Table S8, Table S9.

### Functional relationships

Analysis of transcriptional regulation using the PASTAA algorithm (Roider et al. 2007) indicated that expression of four of these five genes (ACAA1, AQP7, ME1, and SLC27A1) is controlled by a common transcription factor, early growth response (EGR‐4), hypergeometric *P*‐value 7.7 × 10^−4^.

**Figure 4:**
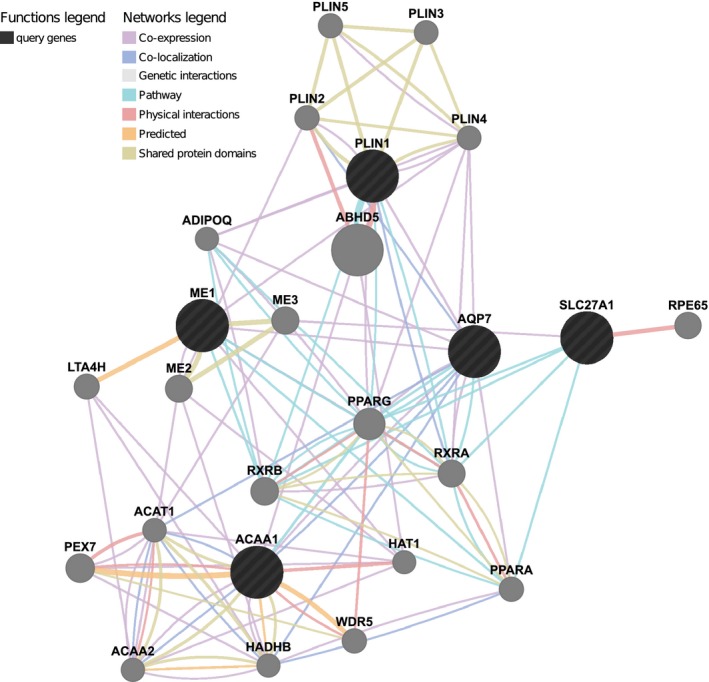
Functional gene relationships based on protein–protein, protein–DNA, and genetic interactions, pathways, reactions, gene and protein expression data, and protein domains. Gene function predictions are based on gene ontology (GO) annotations patterns.

The relationships between the five genes highlighted by GGGL were further characterized using the GeneMANIA prediction algorithm (Warde‐Farley et al. 2010), revealing close coexpression links as well as physical interactions between the seed genes and additional interacting genes (Fig. 4). This interacting set of 25 genes is significantly enriched for disease associations with fatty liver, hypertriglyceridemia, obesity, insulin resistance, and type 2 diabetes, adjusted *P*‐value < 5 × 10^−6^ (WEBGestalt) (Wang et al. 2013).

## Discussion

This integrated analysis of multivariate imaging and genetic data suggests a relationship between lipid pathways, PPAR signaling particularly, and variability in preterm white matter development. Functional network methods draw a specific focus to a subset of five genes within the PPAR pathway (ACAA1, AQP7, PLIN1, ME1, and SLC27A1), four of which are under common transcriptional control by EGR‐4.

The focus of this study is on white matter development among preterm infants, and in this population TBSS detects variability in white matter features that are related both to adverse perinatal events and later neurodevelopment (Counsell et al. 2008; Eikenes et al. 2011; van Kooij et al. 2012). This provides a basis for within‐group comparison of preterm infants; indeed healthy preterm infants are the best‐matched controls for preterm infants with less favorable features. However, this design prevents inferences about differences between preterm and term infants. Nevertheless, we have detected a possible relationship in brain development that replicates findings in adults, associating lipid metabolism with variation in white matter FA in healthy adults (Braskie et al. 2011; Heise et al. 2011).

Anticipated main effects in the model were gestational age (GA) at birth, which can be viewed as degree of prematurity and length of early separation from the placenta, and postmenstrual age at scan (PMA), which captures ongoing development ex utero. It is known that FA increases in white matter as part of ongoing development in term and preterm infants (Huppi et al. 1998; Neil et al. 1998; Drobyshevsky et al. 2005; Smyser et al. 2015), and therefore PMA was included as a covariate throughout the analysis, in an attempt to focus on variability due to factors other than development. Regarding the implications of these observations for white matter development in later life, alterations of white matter including reduced FA have been shown to persist into adulthood in very preterm individuals and are associated with cognitive function (Allin et al. 2011). Given that 60% of the variability in d‐MRI measures between individuals in the neonatal period can be attributed to genetic factors (Geng et al. 2012), and that this heritability persists into adulthood (Shen et al. 2014) we would hypothesize that a predictive relationship identified in the neonatal period would be preserved or even increased in adults (Trzaskowski et al. 2014). It has also been found in healthy adults aged 20–78 years that the genetic effects of the ApoE4 allele on white matter FA were preserved independently of age (Heise et al. 2011). To evaluate whether variation in biological pathways is associated with white matter structure as a function of degree of prematurity, we firstly allowed GA to remain in the model while adjusting for PMA. The impact of lipid pathways is substantial in this model, as indicated by the significant over‐representation of lipids among the top ranked pathways. When GA is adjusted for alongside PMA, specific lipid pathways such as PPAR signaling and glycerolipid metabolism increase in their degree of importance, although lipid pathways as a group are no longer over‐represented.

A possible interpretation of these findings is that interindividual genetic variability in lipid metabolism has an effect on white matter structure (FA) and this is linked to degree of prematurity (GA). On adjustment for GA the main remaining effects are likely to be genetic influence and environmental variables. In this latter scenario, key lipid metabolic pathways remain an important determinant of white matter integrity in the context of a greater variety of biological processes. It is tenable that the genes highlighted here are involved in processes contributing to normal white matter myelination, and that the disruption of these physiological processes in a subset of preterms contributes to the observed variability in FA within the preterm group.

Multiple sources of evidence indicate that a well‐balanced and carefully timed fatty acid supply during the neonatal period is a determinant of growth, visual development, and cognitive development (Fleith and Clandinin 2005; Innis 2007), although there is active debate on how this should be addressed clinically (Adamkin and Radmacher 2014). We have previously highlighted a member of the PPAR pathway (fatty acid desaturase 2, FADS2) in a separate candidate gene analysis of this cohort (Boardman et al. 2014). FADS polymorphisms have since been independently associated with behavioral outcomes in children, suggesting a programming effect of PPAR genotype (Jensen et al. 2014). Other systemic effects of FADS2 could be mediated via its role in catalyzing the conversion of linoleic acid (LA) into arachidonic acid (AA) and that of alpha‐linolenic acid (ALA) into eicosapentaenoic acid (EPA). Polymorphisms in FADS1 and FADS2 have been linked to a proinflamatory phenotype promoting atherosclerosis and coronary artery disease (Martinelli et al. 2008; Glaser et al. 2011), and there appears to be a genetic regulation of the level of desaturase activity that varies with ethnicity (Merino et al. 2011; Sergeant et al. 2012; Chilton et al. 2014).

The peroxisome proliferator‐activated receptors (PPARs) are ligand‐activated transcription factors belonging to the superfamily of nuclear hormone receptors, with an important role in nutrient homeostasis. They are involved in cell membrane structure, signaling, inflammation and biotransformation (Rosen and Spiegelman 2001), neuronal and glial differentiation and axon polarity, and neuroprotection (Gray et al. 2011; Minghetti et al. 2014; Quintanilla et al. 2014). Preterm birth is associated with an increased cardiometabolic disease risk in adulthood (Ryckman et al. 2013; Bayman et al. 2014; Kajantie and Hovi 2014), and PPAR‐gamma agonists (thiazolidinediones) are widely used in the treatment of Type 2 Diabetes Mellitus (T2DM) and insulin resistance. T2DM features here among the top 30 pathways adjusted for GA and PMA, and four of the five PPAR pathway genes highlighted by GGGL are linked to T2DM.

ME1 has been associated with sex‐specific gene regulation in the offspring as a result of peri‐conception maternal obesity (Dahlhoff et al. 2014), and identified as a key regulator of a T2DM‐specific gene expression network (Zhong et al. 2010). PLIN1 regulates droplet formation in lipopolysaccharide‐stimulated microglia (Khatchadourian et al. 2012) and mutations have been linked to familial lypodistrophy and severe insulin resistance and T2DM (Kozusko et al. 2015). SLC27A1 (also known as fatty acid transport molecule 1, FATP1) is localized to mitochondria (Guitart et al. 2014), is involved in fatty acid transport across the blood–brain barrier (Mitchell et al. 2011) and has been considered as a therapeutic target for insulin resistance (Matsufuji et al. 2012). There are indications that AQP7 expression is associated with insulin resistance and obesity (Lebeck 2014), and missense mutations in humans result in a variety of neurological sequelae including severe hypotonia, psychomotor retardation and/or epilepsy as well as multisystem abnormalities (Goubau et al. 2013). ACAA1 is involved in neuronal growth and myelinogenesis (Houdou et al. 1993) and may modify immune responses via Toll‐like signaling (Sordillo et al. 2011).

ACAA1, AQP7, ME1, and SLC27A1 are jointly regulated by the EGR‐4 transcription factor, which is important in neuronal maturation (Ludwig et al. 2011) and synaptic plasticity (Beckmann and Wilce 1997). EGR4 gene expression is induced by cerebral ischemia and inflammation (Decker et al. 2003; Mengozzi et al. 2012), key mechanisms in preterm brain injury (Vannucci and Hagberg 2004; Volpe 2012). EGR4 is also upregulated by EGFR signaling (Mayer et al. 2009), linked to myelination and remyelination (Aguirre et al. 2007). Both fatty acids and EGR signaling have been associated with mental illnesses including schizophrenia (Yamada et al. 2007; Matsumata et al. 2014) and the interaction of EGR‐1 and the PPAR pathway has been described in relation to cardiovascular risk (Fruchart 2009).

### Additional considerations

In this analysis we have performed a within‐group comparison, using the contrast between preterm infants with different values of white matter FA to highlight differences in genetic profile that could add to the biological understanding of white matter development following premature birth. This is driven in part by the paucity of comparable linked imaging and genetic data from healthy term infants, but as a corollary allows us to specifically ask why preterm infants of a similar gestation and at a similar stage of postnatal development have different white matter features that are expected to be functionally relevant. Adjustment for genetic ancestry appeared to leave no independent associations detected by the PsRRR algorithm, suggesting a role for ethnicity and genetic background (the complete genotype of an organism across all loci) in modifying the phenotypic consequences of an allele and its association with disease risk. The impact of alleles may be modified by the local genetic architecture (Jing et al. 2014), and it could be that the effect of ancestry is acting through differences in lipid metabolism. In view of the small sample size involved it would be beyond the scope of this work to make statements about the significance of this observation, but this suggests an exciting avenue for further study.

## Conclusion

In this hypothesis generating work, interpretation of our findings suggests that genetic variation in lipid pathways might influence white matter development among preterm infants. The relationship between imaging measures of brain development and genetic profile appears to be modulated by degree of prematurity. Given that preterm infants are at increased risk of both mental illness and cardiovascular morbidity in later life, this work suggests a unifying mechanism through which these systemic effects might be mediated.

## Conflict of Interest

None declared.

## Supporting information


**Figure S1.** Scree plots of PCA for TBSS phenotype adjusted for PMA (left) and GA plus PMA (right).
**Figure S2**. Frequency density distributions of residuals in TBSS phenotypes adjusted for PMA, GA and PMA, or GA, PMA, and ethnicity.
**Figure S3**. First two components from principal component analysis of population stratification based on pairwise identity by state (IBS).
**Table S4**. Mapping of SNPs to genes and pathways.
**Figure S5**. Null selection frequencies for all KEGG pathways in the PsRRR model.
**Table S6**. PsRRR pathway rankings with null and empirical selection frequencies adjusted for PMA.
**Table S7**. PsRRR pathway rankings with null and empirical selection frequencies adjusted for GA and PMA.
**Table S8**. GGGL‐1 gene selection frequencies >0.4.
**Table S9**. GGGL‐2 top thirty SNPs.
