# Supplementary material for: Possible relationship between common genetic variation and white matter development in a pilot study of preterm infants
Source: Brain Behav. 2016 Apr 2;6(7):e00434. doi: 10.1002/brb3.434 (PMC4821839; doi:10.1002/brb3.434)
Supplement: Supplementary file 1 — Figure S1. Scree plots of PCA for TBSS phenotype adjusted for PMA (left) and GA plus PMA (right). Figure S2. Frequency density distributions of residuals in TBSS phenotypes adjusted for PMA, GA and PMA, or GA, PMA, and ethnicity. Figure S3. First two components from principal component analysis of population stratification based on pairwise identity by state (IBS). Table S4. Mapping of SNPs to genes and pathways. Figure S5. Null selection frequencies for all KEGG pathways in the PsRRR model. Table S6. PsRRR pathway rankings with null and empirical selection frequencies adjusted for PMA. Table S7. PsRRR pathway rankings with null and empirical selection frequencies adjusted for GA and PMA. Table S8. GGGL‐1 gene selection frequencies >0.4. Table S9. GGGL‐2 top thirty SNPs. [file BRB3-6-e00434-s001.docx]

**Supplementary information**

Fig. S1. Scree plots of PCA for TBSS phenotype adjusted for PMA (left) and GA plus PMA (right)

**
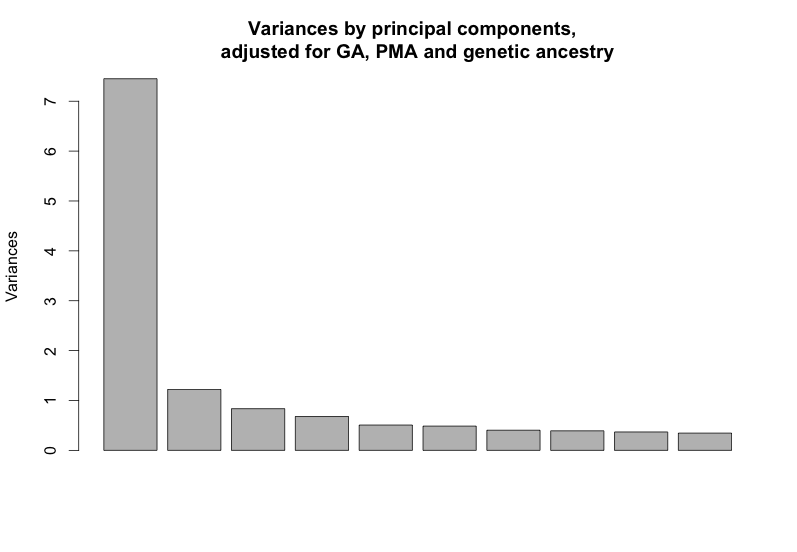
**

The first three components (explaining 47% of total variance) were retained from the phenotype adjusted for PMA, and the first two components were retained from the phenotype adjusted for GA and PMA, as well as from the phenotype adjusted for GA, PMA and genetic ancestry, accounting for 39% of total variance in both cases.

Fig. S2. Frequency density distributions of residuals in TBSS phenotypes adjusted for PMA, GA and PMA, or GA, PMA and ethnicity.


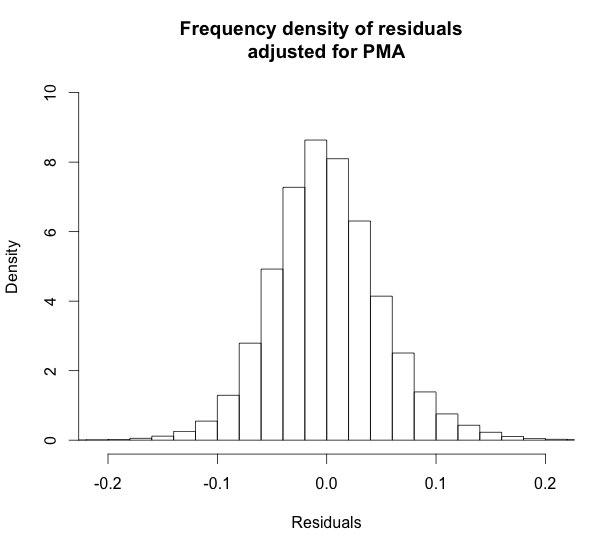

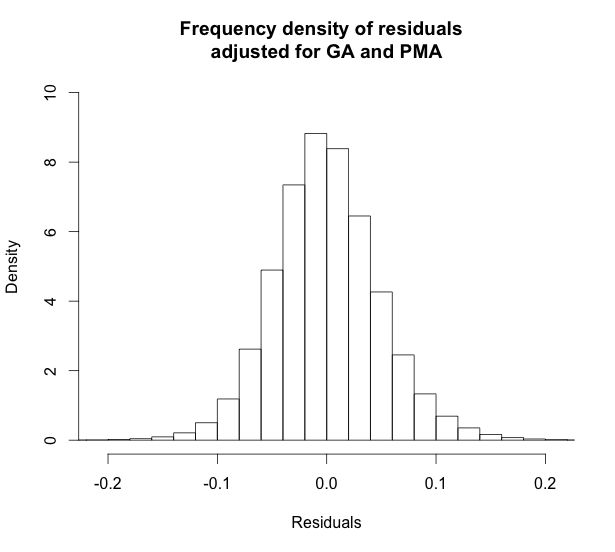


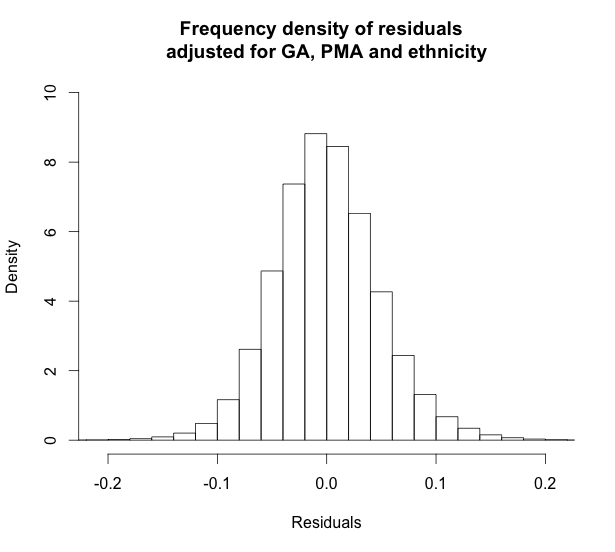


Fig. S3. First two components from principal component analysis of population stratification based on pairwise identity by state (IBS). Points coloured by parental self-reported ethnicity.


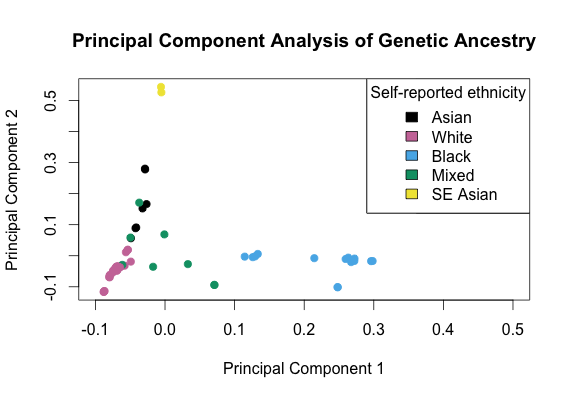


Table S4. Mapping of SNPs to genes and pathways

| **Pathways (total)** | 186 |
| --- | --- |
| **Genes (total)** | 30717 |
| **Genes mapped to pathways** | 5270 |
| **SNPs mapped to genes** | 370774 |
| **SNPs mapped to pathways** | 104229 |
| **Genes mapped to SNPs to pathways** | 5102 |

Fig. S5. Null selection frequencies for all KEGG pathways in the PsRRR model. Left: Model adjusted for PMA. Iteration 20/20, with λ 0.99, 100 subsamples, 20 iterations with 2000x10 plus 4000x10 model fits per iteration. Right: Model adjusted for GA and PMA. Iteration 16/20, with λ 0.99, 100 subsamples, 20 iterations with 2000x10 plus 4000x10 model fits per iteration.


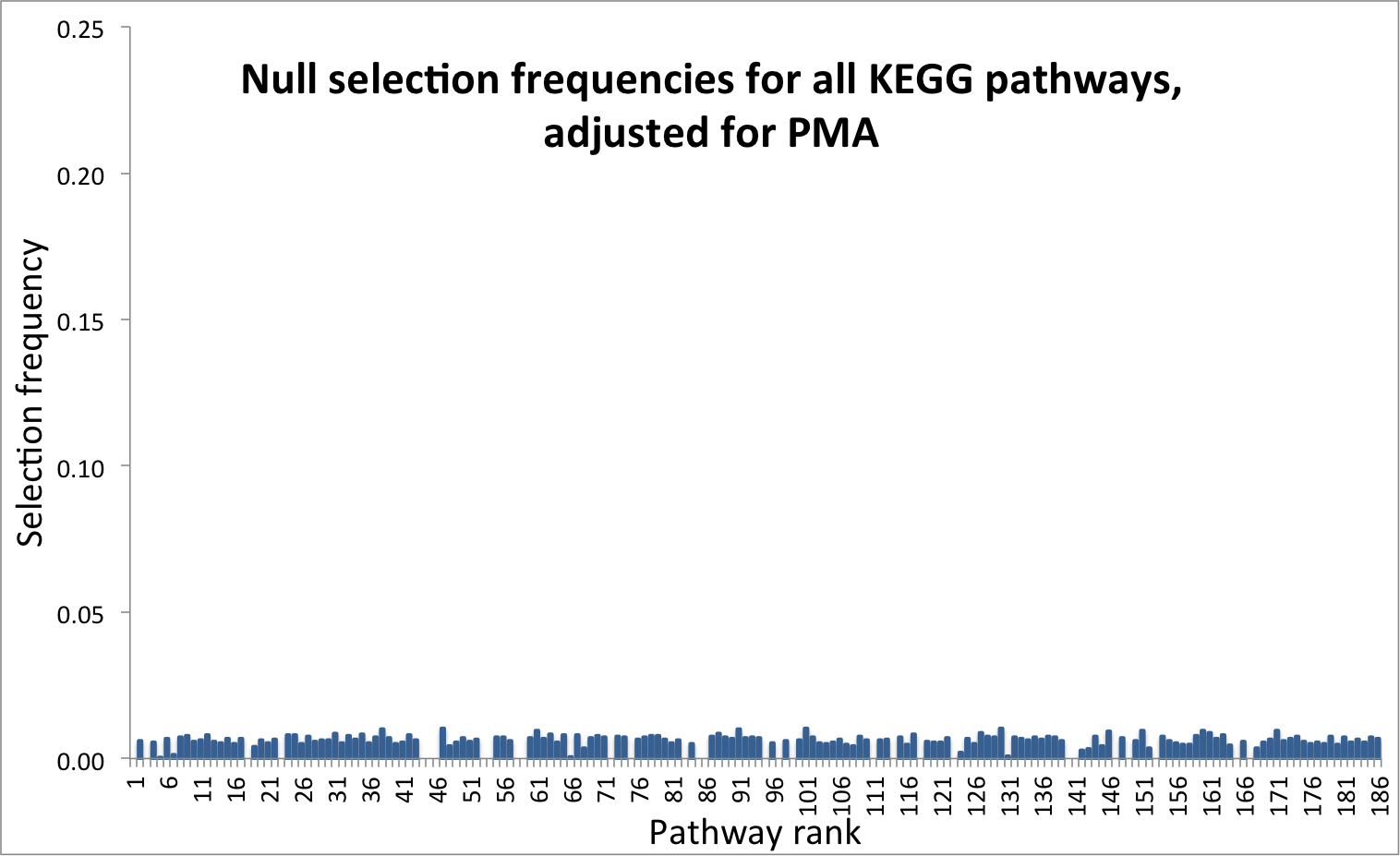

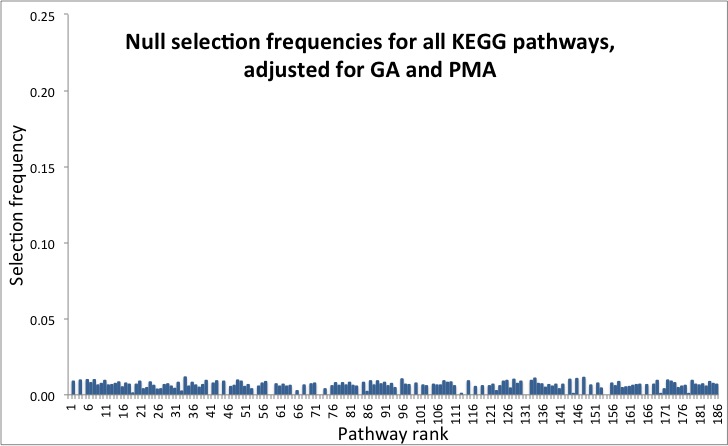


Table S6. PsRRR pathway rankings with null and empirical selection frequencies adjusted for PMA.

| **Rank** | **Pathways adjusted for PMA** | **Empirical selection frequency PMA** | **Null selection frequency** |
| --- | --- | --- | --- |
| 1 | Glycine Serine And Threonine Metabolism | 0.09 | 0.00 |
| 2 | Ppar Signaling Pathway | 0.09 | 0.01 |
| 3 | Alpha Linolenic Acid Metabolism | 0.08 | 0.00 |
| 4 | Ether Lipid Metabolism | 0.08 | 0.01 |
| 5 | Glycerophospholipid Metabolism | 0.07 | 0.00 |
| 6 | Snare Interactions In Vesicular Transport | 0.05 | 0.01 |
| 7 | Hypertrophic Cardiomyopathy Hcm | 0.05 | 0.00 |
| 8 | Glycerolipid Metabolism | 0.05 | 0.01 |
| 9 | Basal Transcription Factors | 0.05 | 0.01 |
| 10 | Cardiac Muscle Contraction | 0.04 | 0.01 |
| 11 | Hematopoietic Cell Lineage | 0.04 | 0.01 |
| 12 | Phosphatidylinositol Signaling System | 0.03 | 0.01 |
| 13 | Ubiquitin Mediated Proteolysis | 0.03 | 0.01 |
| 14 | Nucleotide Excision Repair | 0.03 | 0.01 |
| 15 | Jak Stat Signaling Pathway | 0.02 | 0.01 |
| 16 | Adipocytokine Signaling Pathway | 0.02 | 0.01 |
| 17 | Glycosylphosphatidylinositol Gpi Anchor Biosynthesis | 0.02 | 0.01 |
| 18 | Gnrh Signaling Pathway | 0.02 | 0.00 |
| 19 | Starch And Sucrose Metabolism | 0.02 | 0.00 |
| 20 | Long Term Depression | 0.02 | 0.01 |
| 21 | Abc Transporters | 0.02 | 0.01 |
| 22 | Endocytosis | 0.02 | 0.01 |
| 23 | Fatty Acid Metabolism | 0.01 | 0.00 |
| 24 | Antigen Processing And Presentation | 0.01 | 0.01 |
| 25 | Ascorbate And Aldarate Metabolism | 0.01 | 0.01 |
| 26 | Lysosome | 0.01 | 0.01 |
| 27 | One Carbon Pool By Folate | 0.01 | 0.01 |
| 28 | Fc Epsilon Ri Signaling Pathway | 0.01 | 0.01 |
| 29 | Viral Myocarditis | 0.01 | 0.01 |
| 30 | Complement And Coagulation Cascades | 0.01 | 0.01 |

Table S7. PsRRR pathway rankings with null and empirical selection frequencies adjusted for GA and PMA.

| **Rank** | **Pathways adjusted for GA and PMA** | **Empirical selection frequency GA PMA** | **Null selection frequency** |
| --- | --- | --- | --- |
| 1 | Ppar Signaling Pathway | 0.2 | 0.00 |
| 2 | Dilated Cardiomyopathy | 0.1 | 0.01 |
| 3 | Glycerolipid Metabolism | 0.09 | 0.00 |
| 4 | Alpha Linolenic Acid Metabolism | 0.04 | 0.01 |
| 5 | Pyrimidine Metabolism | 0.03 | 0.00 |
| 6 | Calcium Signaling Pathway | 0.03 | 0.01 |
| 7 | Cardiac Muscle Contraction | 0.03 | 0.01 |
| 8 | Hematopoietic Cell Lineage | 0.03 | 0.01 |
| 9 | Complement And Coagulation Cascades | 0.03 | 0.01 |
| 10 | Aminoacyl Trna Biosynthesis | 0.03 | 0.01 |
| 11 | Pancreatic Cancer | 0.03 | 0.01 |
| 12 | Renin Angiotensin System | 0.03 | 0.01 |
| 13 | Nucleotide Excision Repair | 0.03 | 0.01 |
| 14 | Snare Interactions In Vesicular Transport | 0.02 | 0.01 |
| 15 | Glycosylphosphatidylinositol Gpi Anchor Biosynthesis | 0.02 | 0.01 |
| 16 | Type Ii Diabetes Mellitus | 0.02 | 0.01 |
| 17 | Epithelial Cell Signaling In Helicobacter Pylori Infection | 0.02 | 0.01 |
| 18 | Dna Replication | 0.02 | 0.01 |
| 19 | Glycine Serine And Threonine Metabolism | 0.02 | 0.00 |
| 20 | Sulfur Metabolism | 0.02 | 0.01 |
| 21 | Dorso Ventral Axis Formation | 0.02 | 0.01 |
| 22 | Peroxisome | 0.02 | 0.00 |
| 23 | Bladder Cancer | 0.02 | 0.00 |
| 24 | Primary Immunodeficiency | 0.01 | 0.01 |
| 25 | Ascorbate And Aldarate Metabolism | 0.01 | 0.01 |
| 26 | Lysosome | 0.01 | 0.00 |
| 27 | One Carbon Pool By Folate | 0.01 | 0.00 |
| 28 | Axon Guidance | 0.01 | 0.01 |
| 29 | Vascular Smooth Muscle Contraction | 0.01 | 0.01 |
| 30 | Fc Epsilon Ri Signaling Pathway | 0.01 | 0.01 |

Table S8. GGGL-1 gene selection frequencies > 0.4.

| **SNP Names** | **Gene mapping** | **Selection frequency** |
| --- | --- | --- |
| rs3758267 | AQP7 | 0.855 |
| rs4879696 | AQP7 | 0.817 |
| rs1143796 | ME1 | 0.684 |
| rs1535588 | ME1 | 0.684 |
| rs1170348 | ME1 | 0.683 |
| rs1144184 | ME1 | 0.682 |
| rs9449593 | ME1 | 0.659 |
| rs3798890 | ME1 | 0.648 |
| rs6917851 | ME1 | 0.634 |
| rs12191369 | ME1 | 0.634 |
| rs1180242 | ME1 | 0.607 |
| rs1180192 | ME1 | 0.593 |
| rs7169981 | PLIN1 | 0.553 |
| rs11073884 | PLIN1 | 0.553 |
| rs2289487 | PLIN1 | 0.552 |
| rs8179043 | PLIN1 | 0.546 |
| rs12351969 | AQP7 | 0.537 |
| rs6790738 | ACAA1 | 0.533 |
| rs12630114 | ACAA1 | 0.532 |
| rs7744 | ACAA1 | 0.527 |
| rs6512198 | SLC27A1 | 0.515 |
| rs11086076 | SLC27A1 | 0.515 |
| rs9825655 | ACAA1 | 0.513 |
| rs2278280 | SLC27A1 | 0.511 |
| rs11665931 | SLC27A1 | 0.506 |
| rs4808657 | SLC27A1 | 0.49 |
| rs11668681 | SLC27A1 | 0.483 |
| rs7255307 | SLC27A1 | 0.465 |
| rs9311180 | ACAA1 | 0.459 |
| rs1954537 | ME1 | 0.458 |
| rs4808652 | SLC27A1 | 0.455 |
| rs6599263 | ACAA1 | 0.448 |
| rs1123569 | ACAA1 | 0.438 |
| rs13219666 | ME1 | 0.422 |
| rs11670276 | SLC27A1 | 0.415 |
| rs10890467 | CYP4A22 | 0.409 |
| rs750385 | ME1 | 0.403 |
| rs11666579 | SLC27A1 | 0.403 |

Table S9. GGGL-2 top thirty SNPs.

| **SNP Names** | **Gene mapping** | **Selection frequency** |
| --- | --- | --- |
| rs3758267 | AQP7 | 0.85 |
| rs4879696 | AQP7 | 0.746 |
| rs1143796 | ME1 | 0.718 |
| rs1170348 | ME1 | 0.718 |
| rs1535588 | ME1 | 0.716 |
| rs1144184 | ME1 | 0.712 |
| rs9449593 | ME1 | 0.686 |
| rs1180242 | ME1 | 0.65 |
| rs7169981 | PLIN1 | 0.548 |
| rs11073884 | PLIN1 | 0.548 |
| rs2289487 | PLIN1 | 0.546 |
| rs8179043 | PLIN1 | 0.538 |
| rs6512198 | SLC27A1 | 0.538 |
| rs11086076 | SLC27A1 | 0.528 |
| rs2278280 | SLC27A1 | 0.524 |
| rs11665931 | SLC27A1 | 0.516 |
| rs1180192 | ME1 | 0.506 |
| rs11668681 | SLC27A1 | 0.498 |
| rs10890467 | CYP4A22 | 0.498 |
| rs3798890 | ME1 | 0.492 |
| rs7255307 | SLC27A1 | 0.48 |
| rs4808652 | SLC27A1 | 0.462 |
| rs6790738 | ACAA1 | 0.458 |
| rs12630114 | ACAA1 | 0.458 |
| rs4808657 | SLC27A1 | 0.448 |
| rs7744 | ACAA1 | 0.442 |
| rs6917851 | ME1 | 0.44 |
| rs12191369 | ME1 | 0.44 |
| rs9825655 | ACAA1 | 0.43 |
| rs11670276 | SLC27A1 | 0.426 |
